# Prolactin signaling through focal adhesion complexes is amplified by stiff extracellular matrices in breast cancer cells

**DOI:** 10.18632/oncotarget.10137

**Published:** 2016-06-17

**Authors:** Craig E. Barcus, Patricia J. Keely, Kevin W. Eliceiri, Linda A. Schuler

**Affiliations:** ^1^ Department of Comparative Biosciences, University of Wisconsin-Madison, Madison, WI 53706, USA; ^2^ Cellular and Molecular Biology Program, University of Wisconsin-Madison, Madison, WI 53706, USA; ^3^ Department of Cell and Regenerative Biology, University of Wisconsin-Madison, Madison, WI 53706, USA; ^4^ Laboratory for Cellular and Molecular Biology and Laboratory for Optical and Computational Instrumentation, University of Wisconsin-Madison, Madison, WI 53706, USA; ^5^ University of Wisconsin Paul P. Carbone Comprehensive Cancer Center, University of Wisconsin-Madison, Madison, WI 53706, USA

**Keywords:** prolactin, desmoplasia, breast cancer, extracellular matrix, tumor progression

## Abstract

Estrogen receptor α positive (ERα+) breast cancer accounts for most breast cancer deaths. Both prolactin (PRL) and extracellular matrix (ECM) stiffness/density have been implicated in metastatic progression of this disease. We previously demonstrated that these factors cooperate to fuel processes involved in cancer progression. Culture of ERα+ breast cancer cells in dense/stiff 3D collagen-I matrices shifts the repertoire of PRL signals, and increases crosstalk between PRL and estrogen to promote proliferation and invasion. However, previous work did not distinguish ECM stiffness and collagen density. In order to dissect the ECM features that control PRL signals, we cultured T47D and MCF-7 cells on polyacrylamide hydrogels of varying elastic moduli (stiffness) with varying collagen-I concentrations (ligand density). Increasing stiffness from physiological to pathological significantly augmented PRL-induced phosphorylation of ERK1/2 and the SFK target, FAK-Y925, with only modest effects on pSTAT5. In contrast, higher collagen-I ligand density lowered PRL-induced pSTAT5 with no effect on pERK1/2 or pFAK-Y925. Disrupting focal adhesion signaling decreased PRL signals and PRL/estrogen-induced proliferation more efficiently in stiff, compared to compliant, extracellular environments. These data indicate that matrix stiffness shifts the balance of PRL signals from physiological (JAK2/STAT5) to pathological (FAK/SFK/ERK1/2) by increasing PRL signals through focal adhesions. Together, our studies suggest that PRL signaling to FAK and SFKs may be useful targets in clinical aggressive ERα+ breast carcinomas.

## INTRODUCTION

Estrogen receptor alpha positive (ERα+) breast cancers constitute the most plentiful breast cancer subtype [1], and metastatic ERα+ tumors result in the majority of patient mortality [2, 3]. Although estrogen and progesterone actions in this disease have been the focus of considerable study, the role of prolactin (PRL) remains poorly understood. PRL is best known as a pituitary hormone, but it is also produced locally in multiple tissues, including the breast [4]. Together with ovarian steroid hormones, it drives development and differentiation of lobuloalveoli during pregnancy, chiefly through the Janus Kinase 2 (JAK2)-Signal Transducer and Activator of Transcription 5 (STAT5) signaling cascade [5–7]. Recent large epidemiologic studies have correlated elevated exposure to PRL with increased risk for development of aggressive ERα+ cancers [8, 9]. However, its actions in established cancers are unclear. While some small studies have linked increased PRL/PRL receptor (PRLR) expression to metastasis, therapeutic resistance and poor survival [10, 11], activation of STAT5 correlates with well-differentiated luminal tumors and favorable patient outcomes [12–14].

The discrepancies in these studies present an apparent conflict in PRL actions in breast cancer: PRL activity has been correlated with aggressive ERα+ tumors, yet activation of the canonical PRL signaling mediator correlates with favorable outcomes. A possible explanation may be that PRL can also signal through other effectors, including Focal Adhesion Kinase (FAK), Src-Family Kinases (SFKs), and ERK1/2 [15–17], which may enable aggressive luminal cancers to co-opt PRL signals for pro-tumorigenic purposes. Little is known about the factors that determine the relative strengths of PRL signals to STAT5 and non-canonical pathways; however, one factor that may alter the balance of PRL signals is the extracellular matrix (ECM).

The ECM is increasingly recognized as an active participant in breast cancer. Increased mammographic density, which is comprised of both increased cellular density and fibrillar collagen, correlates with increased breast cancer risk [18–20]. Increased mammographic density also correlates with elevated circulating PRL [21, 22]. Breast carcinomas express higher levels of collagen-I than normal breast tissue and ductal carcinoma *in situ* [23], and the matrix that they encounter during invasion is abundant in fibrillar collagens such as collagen-I [24]. One of the hallmarks of aggressive tumors is desmoplasia [25, 26], which is associated with stiffening of the ECM in and around the primary tumor. Stiffening of the ECM increases formation of focal adhesions and invasion of tumor epithelia, and decreases responsiveness to therapy [27, 28]. Organization of the ECM also correlates with reduced survival, particularly in ERα+ breast cancers [29].

Utilizing a 3-dimensional floating collagen-I gel *in vitro* system [30], we recently reported that high density/stiff collagen environments shift the balance of PRL signals from pSTAT5 to pERK1/2 by activation of the FAK-SFK signaling cascade [31]. Additionally, this environment increases pro-tumor progressive PRL and estrogen cross-talk through SFKs [32]. PRL signals to normal mammary epithelial cells are regulated in part by β1-integrin signals through integrin-like kinase, which enhances PRL signals to pSTAT5 (reviewed in [33]). However, when normal mammary epithelial cells are cultured on collagen-I, PRL signals to pSTAT5 and milk protein expression are decreased [34]. These data indicate that ECM regulation of PRL signals is dependent on both cell phenotype and the properties of the surrounding ECM. Matrix stiffness and high collagen density, which also increases cell surface-bound ligand, are frequently linked. However, during pregnancy, collagen-I is abundant, yet the mammary gland remains compliant and tumor suppressive [35], indicating that matrix stiffness and density of the matrix are distinct properties. Despite the importance of hormones in ERα+ breast cancer, the individual contributions of matrix stiffness and ligand density to endocrine signals in tumor progression remain poorly understood.

To distinguish the impacts of matrix stiffness and ligand density on PRL signals in breast cancer cells, we examined PRL-induced signaling in ERα+, PRLR+ breast cancer cell lines cultured on well-characterized polyacrylamide hydrogels [36, 37] coated with collagen-I [38, 39]. The polyacrylamide hydrogel system decouples matrix stiffness and ligand density, enabling examination of their individual contributions to PRL-induced signals. We report that the rigidity of the ECM modulated PRL signals to FAK/SFK/ERK1/2, while the ligand density of collagen-I was the primary regulator of PRL signals to STAT5. A stiff ECM environment enhanced PRL signals in association with focal adhesions; inhibiting the focal adhesion signaling partners, β1-integrin, FAK, and SFKs, reduced PRL signals to FAK and ERK1/2. Our studies demonstrate that ECM rigidity is a major determinant of PRL signals to the pro-tumor FAK/SFK/ERK1/2 signaling cascade via activation of focal adhesion signaling, elucidating regulation of the downstream signals of PRL and providing a potential therapeutic target in aggressive luminal breast cancers.

## RESULTS

### Increased matrix stiffness increases PRL signals to ERK1/2 and FAK without altering expression of signaling mediators

**Figure 1:**
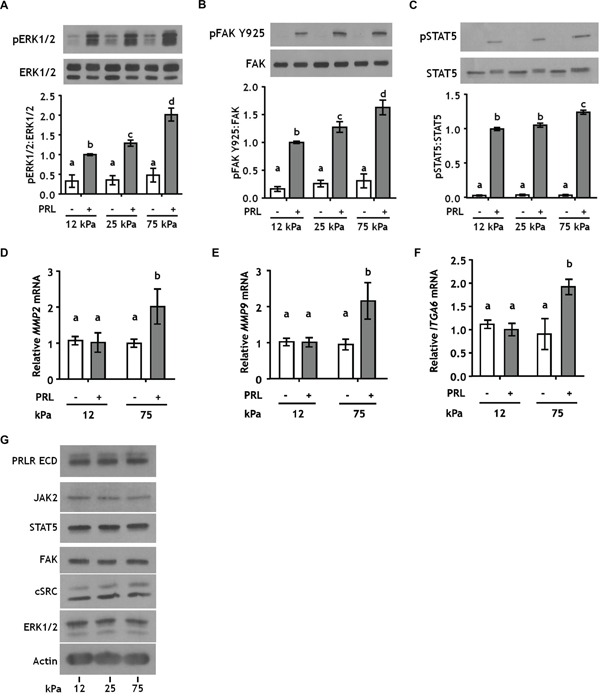
Stiffer environments robustly increase PRL signals to pERK1/2 and pFAK Y925, but only slightly increase signals to pSTAT5 **A-C.** T47D cells were plated on 12, 25, or 75 kPa polyacrylamide gels coated with 200 μg/ml collagen-I, serum starved for 24 h, and treated ± PRL (4nM) for 15 min. Cell lysates were immunoblotted with the indicated antibodies. *Top panels:* Representative immunoblots. *Bottom panels:* Quantification of immunoblots by densitometry. Means ± S.E.M. n = 5. Different letters represent significant differences between treatments, p<0.05. **D-F.** T47D cells were plated and serum starved as in A-C, and treated ± PRL (4nM) for 24h. Specific transcripts were quantitated by RT-PCR as described in the Methods. Means ± S.E.M. n = 3. Different letters represent significant differences between treatments, p<0.05. **G.** T47D cells plated as in A-C were harvested after serum starvation. Cell lysates were immunoblotted with the indicated antibodies.

In a three-dimensional collagen-I environment, we reported that increased stiffness/collagen density increases PRL signals to the FAK/SFK/ERK1/2 cascade in ERα+ breast cancer cells, while decreasing PRL signals to JAK2/STAT5 [31]. However, in the previous system, ligand density and stiffness are interconnected; increasing the density of collagen-I from 1.2 mg/ml (low density/compliant cultures) to 2.8 mg/ml (high density/stiff cultures) also increases the elastic modulus from 12 to 25 kPa, respectively. For comparison, elastic moduli of normal human breast tissue range from 3-20 kPa, ductal carcinoma *in situ* 16-26 kPa, and invasive carcinoma 35-100 kPa [40–42]. In order to isolate the effect of matrix stiffness on PRL-initiated signals, we cultured breast cancer cells on polyacrylamide hydrogels of increasing stiffness, while holding the collagen concentration constant at 200 μg/ml. T47D cells exhibit robust PRL-induced phosphorylation of ERK1/2, FAK, and STAT5 [31]. Stiffer matrices robustly increased PRL-induced signals to the pro-tumorigenic FAK/ERK1/2 cascade (p<0.01) (Figure 1A, 1B; Supplementary Figure 1), while only modestly increasing PRL signals to pSTAT5 in the stiffest matrices (p<0.05) (Figure 1C). This shift in the relative strengths of PRL-induced signaling cascades was reflected in altered transcripts of genes mediating more aggressive behaviors/phenotypes. Transcripts for the matrix metalloproteinases, *MMP2* and *MMP9*, and the progenitor marker *ITGA6* (CD49f) were increased in response to PRL only in the stiff matrix environment (p<0.05) (Figure 1D-1F). Despite these functional changes, matrix stiffness did not alter total protein expression of the PRLR or PRL signaling mediators (Figure 1G), indicating that the observed effects of ECM stiffness are not a result of gross changes in protein expression. Stiffness similarly modulated PRL signals in another luminal breast cancer cell line, MCF-7 cells, confirming this observation across different cell contexts (Supplementary Figure 2).

### Increased collagen-I ligand density decreases PRL signals to pSTAT5, but not pERK1/2 or pFAK Y925

**Figure 2:**
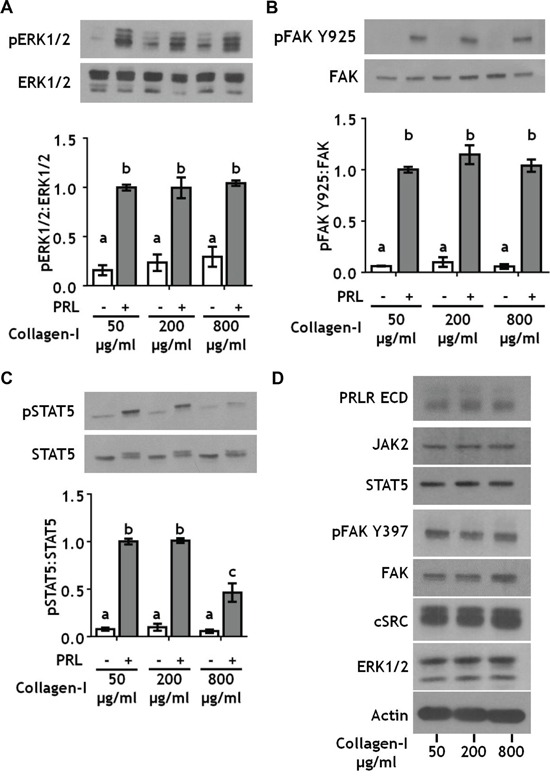
Collagen ligand density does not modulate PRL signals to ERK1/2 or FAK **A-C.** T47D were cells plated on 25 kPa polyacrylamide gels coated with either 50, 200, or 800 μg/ml collagen-I, serum starved for 24h, then treated ± PRL (4 nM) for 15 min. Cell lysates were immunoblotted with the indicated antibodies. *Top panels:* Representative immunoblots. *Bottom panels:* Quantification of immunoblots by densitometry. Means ± S.E.M. n = 3. Different letters represent significant differences between treatments, p<0.05. **D.** T47D cells plated as in *A* were harvested after serum starvation. Cell lysates were immunoblotted with the indicated antibodies.

In order to determine the effect of collagen-I ligand density on PRL-induced signals, we cultured T47D cells on polyacrylamide hydrogels at 25 kPa stiffness and varied the collagen-I ligand concentration from 50 to 800 μg/ml. Altered collagen-I ligand density also did not affect PRL signals to pERK1/2 or pFAK Y925 (Figure 2A, 2B; Supplementary Figure 1). However, high collagen I concentrations (800 μg/ml) significantly reduced PRL signals to pSTAT5 (p<0.01) (Figure 2C). Interestingly, reducing the stiffness of the polyacrylamide matrix to 12 kPa reversed the effect of collagen I concentration on PRL signals to STAT5 (Supplementary Figure 3), suggesting a mechanism for the observations in the pregnant gland [35]. Like altered ECM stiffness, increased collagen-I ligand density did not change total protein expression of PRL signaling mediators (Figure 2D). Additionally, autophosphorylation of FAK at tyrosine 397 (pFAK Y397), one of the major signal transducers of extracellular matrix binding by integrins [43], was saturated at 50 μg/ml collagen-I, indicating that effects on the spectrum of PRL signals are not due to increased FAK activation at this site (Figure 2D). Together, these data indicate that the ligand density of collagen-I controls PRL signals to STAT5. In contrast, the stiffness of the extracellular matrix, not the concentration of collagen-I ligand, controls PRL signals to the pro-tumor progressive FAK/SFK/ERK1/2 signaling cascade.

### β1-integrin enhances PRL signals to pERK1/2 and pFAK Y925 only in stiff environments

**Figure 3:**
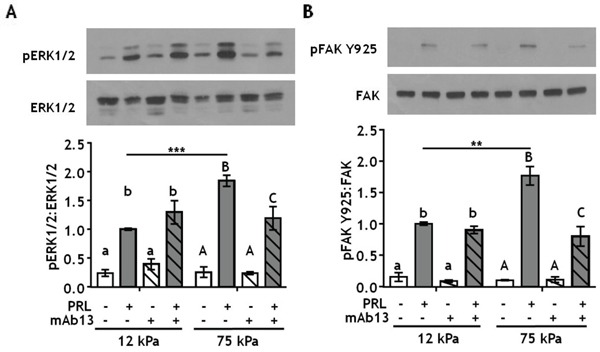
Blocking β1-integrin decreases PRL signals to pERK1/2 and pFAK Y925 in stiff environments **A-B.** T47D cells were plated on 12 or 75 kPa polyacrylamide gels coated with 200 μg/ml collagen-I, serum starved for 24h, then treated with isotype control antibody (−) or β1-integrin blocking antibody mAb13 (+) for 1 h prior to ± PRL (4 nM) for 15 min. Cell lysates were immunoblotted with the indicated antibodies. *Top panels:* Representative immunoblots. *Bottom panels:* Quantification of immunoblots by densitometry. Means ± S.E.M., n = 4. Different letters represent significant differences within each stiffness (lower case, 12kPa; upper case, 75kPa), p<0.05. * represent significant differences between the same treatments at different stiffnesses, *p<0.05, **p<0.01, ***p<0.001.

Stiff extracellular environments increase formation of focal adhesion complexes that contain both FAK and SFKs [44, 45], and growth factor receptor signaling pathways are known components of focal adhesion complexes [46]. Focal adhesions form where clusters of integrin complexes bind to the extracellular matrix, where integrins can regulate growth factor/cytokine receptor signaling (reviewed in [47]). β1-integrin complexes are the major collagen-I receptors on epithelial cells and play key roles in mammary gland development [48, 49] and mammary tumor progression [50, 51]. To determine the effects of matrix stiffness on β1-integrin regulation of PRL signals, T47D cells were cultured on 12 kPa (compliant) or 75 kPa (stiff) hydrogels coated with 200 μg/ml collagen-I and treated ± β1-integrin blocking antibody prior to PRL treatment. Blocking β1-integrin significantly decreased PRL signals to pERK1/2 in a stiff environment, but had no effect in compliant matrices (Figure 3A, p<0.05). PRL signals to FAK Y925 were similarly affected (Figure 3B, p<0.05).

### Inhibiting integrin-activated FAK more potently inhibits PRL signals in stiff environments

**Figure 4:**
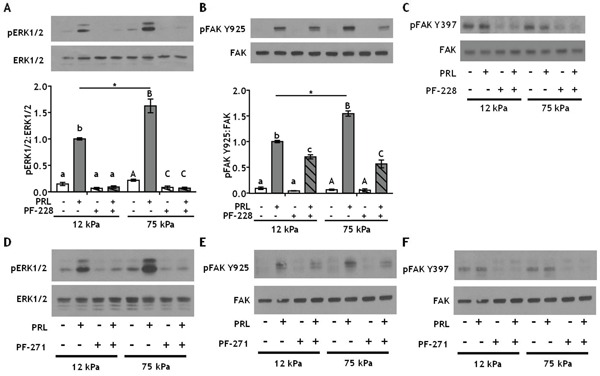
Inhibiting integrin activated FAK at Y397 more efficiently decreases PRL signals to pFAK Y925 in stiff environments **A-B.** T47D cells were plated on 12 or 75 kPa polyacrylamide gels coated with 200 μg/ml collagen-I, serum starved for 24h, then treated with vehicle (−) or FAK Y397 inhibitor PF-573228 (+) for 1 h prior to ± PRL (4 nM) for 15 min. Cell lysates were immunoblotted with the indicated antibodies. *Top panels:* Representative immunoblots. *Bottom panels:* Quantification of immunoblots by densitometry. Means ± S.E.M. n = 4. Different letters represent significant differences within each stiffness (lower case, 12kPa; upper case, 75kPa), p<0.05. * represent significant differences between the same treatments at different stiffnesses, *p<0.05. **C.** T47D cells were plated and treated as in *A*. Cell lysates were immunoblotted with indicated antibodies. **D-F.** T47D cells plated as in *A* were treated with vehicle (−) or the FAK Y397 inhibitor, PF-562271 (+), for 1 h prior to ± PRL (4 nM) for 15 min (representative immunoblots).

Directly downstream of collagen ligand/β1 integrin complexes is FAK, which autophosphorylates at Y397 when integrins engage the ECM [52]. To test whether this FAK autophosphorylation site plays a critical role in the stiffness-modulated PRL signals to the FAK/SFK/ERK1/2 pathway, T47D cells were cultured on 12 or 75 kPa gels coated with 200 μg/ml collagen-I and treated ± the FAK Y397 inhibitor, PF-573228, prior to PRL treatment. Inhibiting pFAK Y397 blocked all PRL signals to pERK1/2 regardless of stiffness (p<0.001) (Figure 4A). In contrast, PF-573228 only slightly reduced PRL signals to pFAK Y925 in compliant matrices, but completely blocked the increased PRL signals in stiff matrices to levels observed in compliant environments (p<0.01) (Figure 4B). Similar results were obtained using another FAK Y397 inhibitor, PF-562271 (Figure 4D-4F).

### Inhibiting SFKs more potently inhibits PRL signals in stiff environments

**Figure 5:**
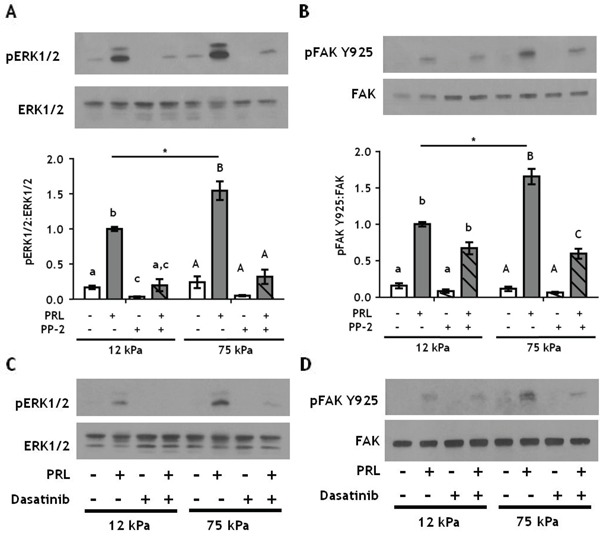
Inhibiting SFKs decreases PRL signals to pFAK Y925 only in stiff environments **A-B.** T47D cells were plated on 12 or 75 kPa polyacrylamide gels coated with 200 μg/ml collagen-I, serum starved for 24h, then treated with vehicle (−) or SFK inhibitor, PP-2 (+) for 1 h prior to ± PRL (4 nM) for 15 min. Cell lysates were immunoblotted with the indicated antibodies. *Top panels:* Representative immunoblots. *Bottom panels:* Quantification of immunoblots by densitometry. Means ± S.E.M. n = 3. Different letters represent significant differences within each stiffness determined by paired t-tests (lower case, 12kPa; upper case, 75kPa), p<0.05. * represent significant differences between the same treatments at different stiffnesses, *p<0.05. **C-D.** T47D cells were plated as in *A* and treated with vehicle (−) or the SFK inhibitor, Dasatinib (+), for 1 h prior to ± PRL (4 nM) for 15 min. Cell lysates were immunoblotted with the indicated antibodies.

SFKs are a family of oncogenes that contribute to progression of breast cancer [53, 54], and are important components of PRL signaling cascades [16, 17]. To test if SFKs mediate effects of PRL in stiff matrices, T47D cells were treated ± PP-2 prior to PRL treatment. Like inhibition of pFAK Y397, PP-2 blocked PRL signals to pERK1/2 regardless of culture stiffness (Figure 5A), and PRL signals to pFAK Y925 were significantly decreased in stiff (p<0.01), but not compliant cultures (Figure 5B). Similar results were obtained using the clinically approved inhibitor, dasatinib (Figure 5C, 5D). These studies indicate that PRL-induced signals to ERK1/2 are under control of the FAK/SFK pathway regardless of ECM stiffness; however, PRL-induced signals to FAK Y925 are not exclusively mediated by SFKs nor are they dependent on pFAK Y397. Other kinases can phosphorylate FAK Y925, including c-MET and BRK (PTK6). However, since the level of PRL-induced pFAK Y925 is equivalent in both compliant and stiff cultures in the presence of FAK inhibitors (Figure 4B, 4E) and SFK inhibitors (Figure 5B, 5D), we conclude that stiffness augmented PRL signals are under the control of FAK and SFK.

### Stiff extracellular environments augment E2/PRL-induced proliferation through FAK

**Figure 6:**
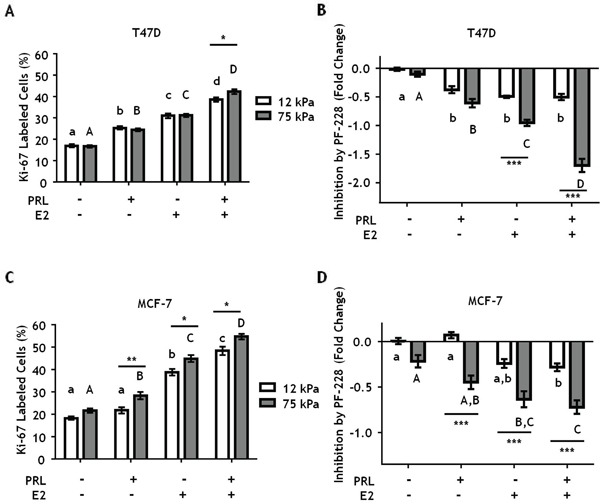
Stiff environments increase FAK-mediated hormone induced proliferation T47D and MCF-7 cells were plated on 12 or 75 kPa polyacrylamide gels coated with 200 μg/ml collagen-I in phenol-red free 5% charcoal stripped FBS for 24 h, serum starved for 24 h, and then treated with vehicle (DMSO 1:1000) or the FAK inhibitor, PF-573228 (1μM), for 1 h prior to ± PRL (4 nM), ± E2 (1nM) for 24 h. Cells were then stained with DAPI and Ki-67 antibody as described in Experimental Procedures. **A, C.** Effect of hormones on Ki67 staining, assessed by percentage of Ki-67 positive T47D (A) and MCF-7 (C) cells. **B, D.** Inhibition of proliferation by PF-573,228 compared to vehicle treated T47D cells (B) and MCF7 cells (D). Different letters represent significant differences within each stiffness (lower case, 12 kPa; upper case, 75 kPa). * represent significant differences between the same treatments at different stiffnesses: *p<0.05, **p<0.01, ***p<0.001.

On 2-D tissue culture plastic, PRL is mitogenic for breast cancer cells [17, 55], and augments estrogen-induced growth [56, 57]. In this extremely stiff environment, PRL activation of FAK through SFKs mediates PRL-induced proliferation [17]. In order to determine if matrix stiffness alters PRL and estrogen-induced proliferation through FAK, T47D cells were plated on hydrogels of different stiffnesses and then treated ± PF-573228 for 1 h prior to hormone treatment for 24 h. Matrix stiffness did not alter PRL or E2-induced proliferation, as indicated by Ki67 labeling, but permitted a small increase in PRL+E2 induced proliferation compared to a compliant environment (p<0.05) (Figure 6A). Inhibiting FAK did not alter proliferation in the absence of hormones, but significantly decreased proliferation in response to hormones in stiff compared to compliant environments, up to 2-fold when both PRL and E2 were present (p<0.001) (Figure 6B). MCF-7 cells displayed a very similar pattern (Figure 6C, 6D). Taken together, these data indicate that stiff extracellular environments enhance PRL signals through focal adhesions, increasing PRL signals to the FAK/SFK/ERK1/2 pro-tumorigenic signaling cascade.

## DISCUSSION

The desmoplastic response during breast cancer progression is well characterized (reviewed in [25, 58]). Deposition of ECM components such as collagen-I increases ligand for cell surface receptors and also physical rigidity, activating mechano-signals through integrin-linked focal adhesions [51]. Physical rigidity, measured by the elastic modulus, is implicated in multiple tumor progressive characteristics such as therapeutic resistance [59], epithelial to mesenchymal transition [60], and increased invasion and aggressiveness [27]. Increasing collagen in breast tissue raises the density of the ECM [20] and correlates with more aggressive tumors [18, 61]. Although our understanding of the individual contributions of PRL and ECM characteristics to breast cancer progression is growing, the relative contributions of physical rigidity and collagen ligand density of the ECM that cooperate with PRL are poorly understood.

**Figure 7:**
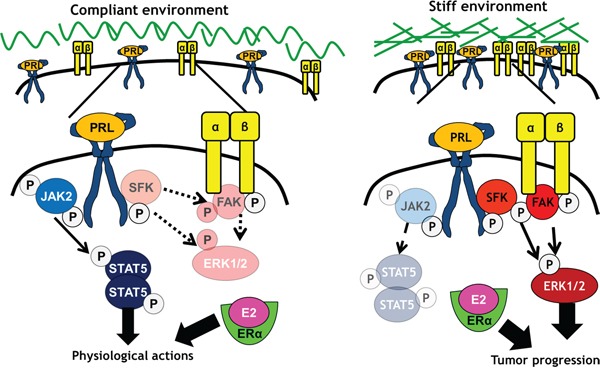
Stiff matrices enhance PRL signals via activation of focal adhesions In compliant environments, PRL/PRLR preferentially activates JAK2/STAT5, with lower activity towards SFKs, FAK, and ERK1/2, resulting in physiological PRL actions. In stiff environments, PRL/PRLR preferentially activates SFKs and FAK, increasing activity towards ERK1/2 and pro-tumor progressive signals and outcomes.

Here we demonstrated that the physical stiffness (elastic modulus) of the ECM, but not collagen I ligand density, controls PRL-induced signals to the pro-tumorigenic FAK/SFK/ERK1/2 signaling pathway, with only modest effects on PRL-induced STAT5 signals. In contrast, collagen density moderates PRL signals to STAT5. Although hormone-induced proliferation was only slightly higher in stiff compared to compliant matrices, the lack of effect of matrix stiffness on PRLR expression suggests a greater portion of these hormonal signals become routed through FAK in stiff matrices, and are more susceptible to inhibition of this pathway. These findings indicate that a stiff extracellular environment promotes PRL signals through focal adhesions, fueling tumor progression (Figure 7). Notably, the breast cancer cell lines examined here are only weakly metastatic *in vivo* [62]. We would predict heightened cooperation between PRL and growth factors through these localized signaling platforms in rigid environments in aggressive luminal B cancers, which also respond strongly to growth factors [46, 47, 63].

Focal adhesion complexes are large sites of cell-matrix interactions, containing numerous receptors, scaffolding proteins, and kinases that transduce extracellular cues to cells [64]. A key component of focal adhesions is FAK, which regulates focal adhesion composition and subsequent cell behavior including motility and tumor cell invasion [45, 65]. Targeting FAK for cancer therapeutics shows promise in pre-clinical animal models, as well as early stage clinical trials (reviewed in [66]). Additionally, SFKs associate with focal adhesions, regulating integrin dynamics [67] and connecting FAK to the MAPK pathway via phosphorylation at Y925 [52, 68]. In ERα+ tumors, FAK and SFKs are implicated in cancer cell invasion [69] and resistance to tamoxifen treatment [54].

Recent evidence indicates that the intracellular domain of PRLR is intrinsically disordered, and can associate with the plasma membrane through distinct lipid modifications [70]. This suggests a mechanism whereby PRLR, in close proximity to membrane bound focal adhesion complexes [31], can activate FAK and SFKs that associate with lipid rich areas [71, 72]. We previously described PRLR and c-Src co-localization in lipid raft microdomains [16], and both lipid raft mediated signals [73] and components [74] associate with aggressive breast cancers in experimental models. Together, these data suggest that PRLR/focal adhesion signals may be enhanced by co-localization of these components in lipid rich areas, leading to activation of pro-tumor progressive signals.

Our findings begin to resolve the apparent dichotomy of PRL actions in breast cancer: PRL can reduce aggressive tumor behavior through STAT5, but increase tumor progression through FAK/SFK/ERK1/2. Activation of the canonical PRL signal mediator, STAT5, is a positive prognostic factor in breast cancer that predicts sensitivity to anti-estrogen therapies and favorable outcomes [12–14]. These relatively differentiated outcomes resemble PRL actions mediated by STAT5 during pregnancy [5–7]. Interestingly, the mammary ECM during pregnancy is compliant, despite the high density of collagen I [35], underscoring the importance of matrix structure, including alignment, composition and crosslinking, in determining matrix stiffness. Increasingly sophisticated imaging and proteomics will enable new insights into the contributions of tumor epithelia and recruited stroma on matrix properties, and consequences for hormone actions. Interestingly, our data indicates that increased collagen density in a relatively compliant matrix does not markedly reduce PRL signals via STAT5 (Supplementary Figure 3). This suggests that these parameters are tightly controlled during pregnancy, and that some tumor environments may retain these matrix features, with more benign outcomes.

We have shown that physical rigidity of the ECM is a major determinant of the spectrum of PRL-induced signals, increasing PRL activation of the tumor progressive FAK/SFK/ERK1/2 signaling cascade in stiff environments through localization of PRLR to focal adhesions. Our data provide a mechanism for how tumor environments can shift PRLR signals away from physiological STAT5, and subsequent positive prognostic outcomes, to the poorer outcomes of increased signals through focal adhesions. Moreover, PRL increases expression of mammary ECM components [75], and increases perpendicularly aligned collagen-I *in vitro* [32], a hallmark of aggressive tumors [29]. A model in which PRL enhances the deposition and reorganization of collagen to increase stiffness, resulting in increased PRL signals in PRLR-focal adhesion complexes, begins to clarify the epidemiologic data, which present PRL as a risk factor for metastatic luminal tumors. Our studies suggest that disrupting PRLR-focal adhesion signals may point to novel therapeutic targets in aggressive ERα+ breast cancers.

## MATERIALS AND METHODS

### Reagents

Recombinant hPRL (Lot AFP795) was obtained from Dr. A.F. Parlow (National Hormone and Pituitary Program, NIDDK, National Institutes of Health, Torrance, CA). Type-I rat tail collagen (#CB354249) was obtained from Fisher Scientific (Pittsburgh, PA). Sulfo-SANPAH (#C1111-100) was obtained from ProteoChem (Indianapolis, IN). Inhibitors used for these studies were purchased as follows: PP-2 (#ab120308) from Abcam (Cambridge, MA), Dasatinib from Selleckchem (Boston, MA), pFAK Y397 inhibitors PF-573228 (#PZ0117) from Sigma Aldrich (St. Louis, MO) and pFAK Y397 inhibitor PF-562271 (#S2890) from Selleck Chemicals (Houston, TX). Protein A/G agarose beads (#SC-2003) were obtained from Santa Cruz Biotechnology (Santa Cruz, CA). Antibodies used in these studies were purchased as follows: PRLR-ECD (#35-9200), pSRC Y418 (#44660G), pFAK Y397 (#44624G), and pSTAT5 (#71-6900) from Invitrogen (Grand Island, NY); ERK1/2 (#9102), pERK1/2 (#9101), FAK (#3285), and pFAK Y925 (#3284) from Cell Signaling Technology (Danvers, MA); cSRC (sc-18), PRLR (sc-20992), and STAT5 (sc-835x) from Santa Cruz Biotechnology (Santa Cruz, CA); FAK clone 4.47 (#05-537) from EMD Millipore (Billerica, MA). Ki-67 (Ab15580) from AbCam (Cambridge, MA); β1-integrin blocking antibody (clone mAb13, cat. # 552828) and rat IgG2a,k isotype antibody (cat. # 555841) was purchased from BD Biosciences (San Jose, CA). Donkey anti-rabbit conjugated to Rhodamine (TRITC) secondary antibody (711-025-152) was purchased from Jackson Immuno-Research (West Grove, PA). All other reagents were obtained from Fisher Scientific or Sigma-Aldrich.

### Polyacrylamide hydrogels

Polyacrylamide gel inserts were prepared as described [36, 37]. Briefly, polyacrylamide gels with elastic moduli of 12, 25, and 75 kPa were made by varying the amount of bisacrylamide present in a 40% acrylamide solution to correspond to the elastic modulus of previously reported three-dimensional collagen gel cultures [32, 44, 76]. Modulus values are reported as elastic (Y) modulus as opposed to shear modulus (G), which has the relationship of Y = 2G(1 + υ) where υ has an approximation of 0.48 for polyacrylamide gels [77]. After hydrating the gels, inserts were trimmed for 12-well tissue culture plates and functionalized with the chemical crosslinker sulfo-SANPAH (2 mg/ml) in distilled, deionized (DI) H_2_O under high intensity UV light for 5 minutes. Excess sulfo-SANPAH was rinsed off in DI H_2_O and collagen-I was then added to the functionalized hydrogels at the appropriate concentrations (50, 200, or 800 μg/ml) [38, 39] for 2 hrs. Highly concentrated rat tail collagen-I was utilized from the same lot throughout all experiments [78]. The gels were then washed in 1X PBS and sterilized under germicidal UV light for 30 minutes prior to the addition of cells.

### Cell culture

ERα^+^, PRLR^+^ T47D and MCF-7 breast cancer cells were maintained as previously described [79, 80]. T47D and MCF-7 cells were plated on functionalized collagen coated polyacrylamide gels at 150,000 cells/well for signaling studies or 75,000 cells/well for proliferation studies. To assess signaling pathways, 72 h after plating, cells were serum starved overnight prior to treatment with PRL (4nM) for 15 min. Immunoblotting of cell lysates was performed as previously described [81]. Briefly, cells were lysed in ice cold modified RIPA buffer containing 1% SDS and phosphatase inhibitors, sheared by needle aspiration, and centrifuged to remove insoluble cell debris prior to fractionation on standard SDS-PAGE gels. Signals were visualized using enhanced chemiluminescence (ThermoFischer), and quantified by scanning densitometry (VisionWorksLS, v7.1, UVP, Upland, CA). To assess gene expression, T47D cells were plated on 12 or 75 kPa polyacrylamide gels coated with 200 μg/ml collagen-I and treated ± PRL for 24 h. RNA was collected with the RNeasy mini-kit (Qiagen, #74104), cDNA synthesized, and quantitative real-time PCR performed as previously described [31]. The following primer sequences were utilized: 18 S F, 5′-CGC CGC TAG AGG TGA AAT TCT; 18 S R, 5′-CGA ACC TCC GAC TTT CGT TCT; *MMP2* F, 5′-CTG CAA CCT GTT TGT GCT GAA; *MMP2* R, 5′-GGC TTG CGA GGG AAG AAG T; *MMP9* F, 5′-CGG AGT GAG TTG AAC CAG; *MMP9* R, 5′-GTC CCA GTG GGG ATT TAC; *ITGA6* F, 5′-CAT ATA GAG AAC TGA GGG CTT TCC; and *ITGA6* R, 5′-TCC GAG CTC ACA GTC AGC TT. For proliferation studies, 24 h after plating in phenol-red free charcoal stripped serum media, cultures were serum starved overnight, and then treated ± 17β-estradiol (E2) 1nM and ± PRL 4nM for 24 h. For some experiments, inhibitors were added 1 h prior to hormone treatment at the following concentrations: 5μM PF-573228, 5 μM PF-562271, 5 μM PP-2, 250 nM dasatinib, or 500 ng/ml mAb13.

### Immunofluorescence

Immunofluorescence was performed as previously described [32]. Briefly, cells cultured on varying stiffness gels were fixed in 4% paraformaldehyde, permeabilized, and blocked in 5% donkey serum, 1% BSA PBS-T overnight at 4C. Cells were incubated with antibody to Ki-67 (1:500) for 1 h at RT followed by extensive washing in PBS-T. Secondary antibody (1:100) and DAPI (1:300) were incubated for 1 h at RT followed by extensive washing. Gels were imaged on a Nikon E600 Eclipse epifluorescent microscope kindly provided by Dr. Chad Vezina. Images were analyzed utilizing the Particle Analysis plugin on ImageJ, examining 5 fields of at least 100 cells per field [82].

### Statistical analyses

Statistical analyses were performed using GraphPad Prism v.4.0. Independent experiments examining signaling cascades by immunoblotting were analyzed via two-way ANOVA followed by post-hoc paired t-tests. Analysis of Ki67 staining for proliferation studies was assessed by two-way ANOVA, followed by Holm-Sidak multiple comparison tests.

## SUPPLEMENTARY FIGURES


