# Supplementary material for: Prolactin signaling through focal adhesion complexes is amplified by stiff extracellular matrices in breast cancer cells
Source: Oncotarget. 2016 Jun 17;7(30):48093–106. doi: 10.18632/oncotarget.10137 (PMC5217003; doi:10.18632/oncotarget.10137)
Supplement: Supplementary file 1 [file oncotarget-07-48093-s001.pdf]

Prolactin signaling through focal adhesion complexes is amplified by stiff extracellular matrices in breast cancer cells

SUPPLEMENTARY FIGURES

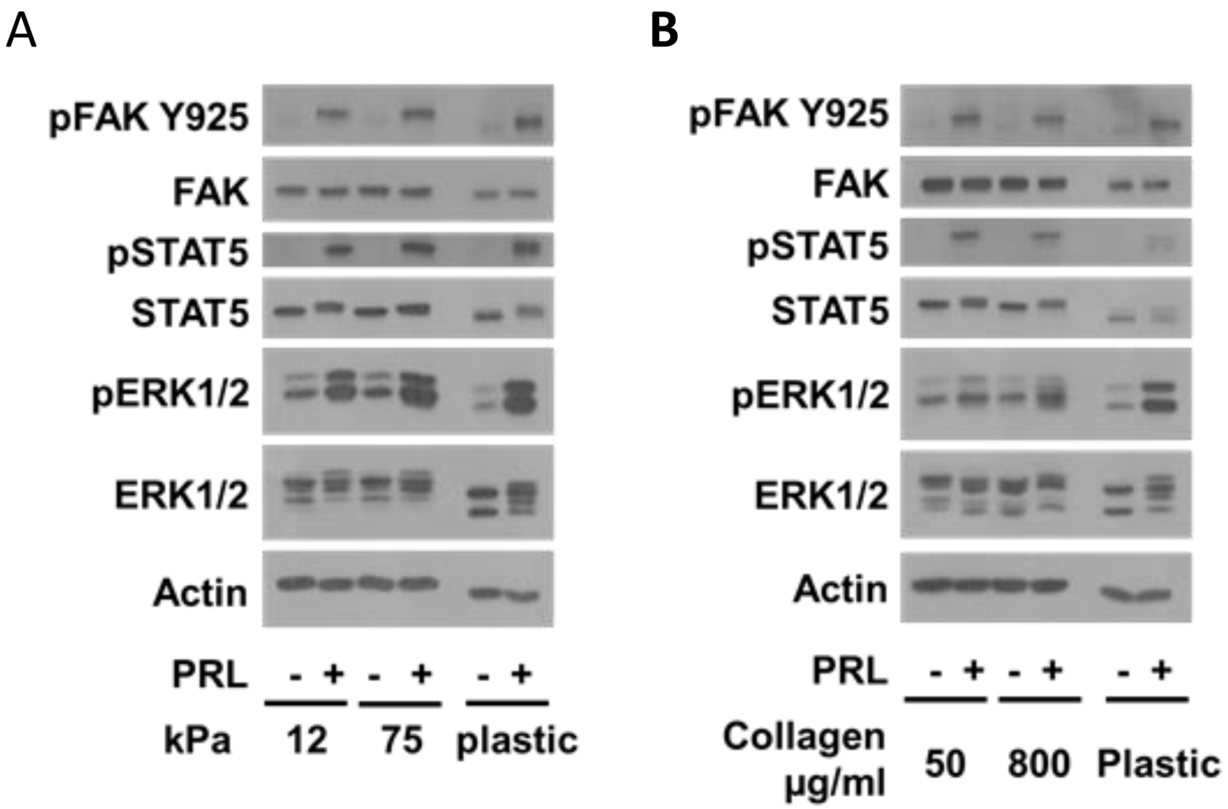

Supplementary Figure S1: A. T47D cells were plated on 12, or 75 kPa polyacrylamide gels coated with 200 µg/ml collagen-I or tissue culture plastic, serum starved for 24h, and then treated ± PRL (4 nM) for 15 min. **B.** T47D cells were plated on 25 kPa polyacrylamide gels coated with 50 or 800 µg/ml collagen-I or tissue culture plastic, serum starved for 24 h, and treated ± PRL (4nM) for 15 min. Cell lysates were immunoblotted with the indicated antibodies.

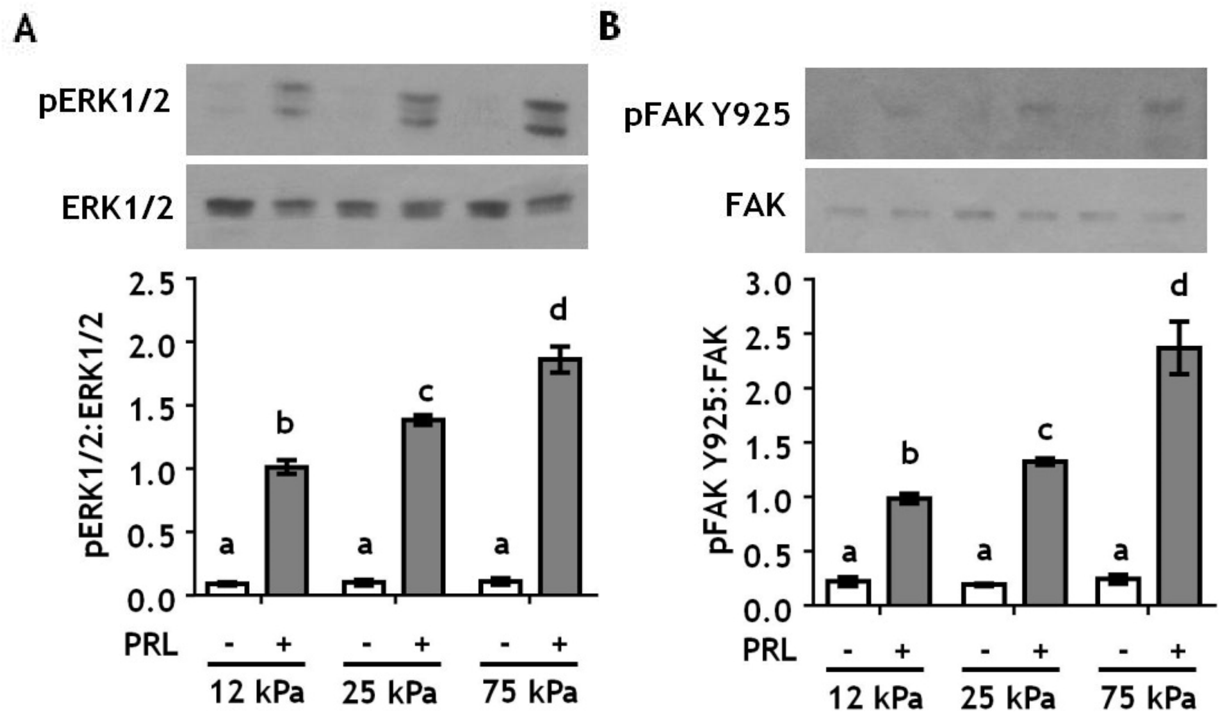

Supplementary Figure S2: MCF-7 cells respond to matrix stiffness mediated PRL-induced signals similarly to T47D cells. A-B. MCF-7 cells were plated on 12, 25, or 75 kPa polyacrylamide gels coated with 200 µg/ml collagen-I, serum starved for 24 h, and treated ± PRL (4nM) for 15 min. Cell lysates were immunoblotted with the indicated antibodies. *Top panels:* Representative immunoblots. *Bottom panels:* Quantification of immunoblots by densitometry. Means ± S.E.M. n = 3. Different letters represent significant differences between treatments, $p<0.05$.

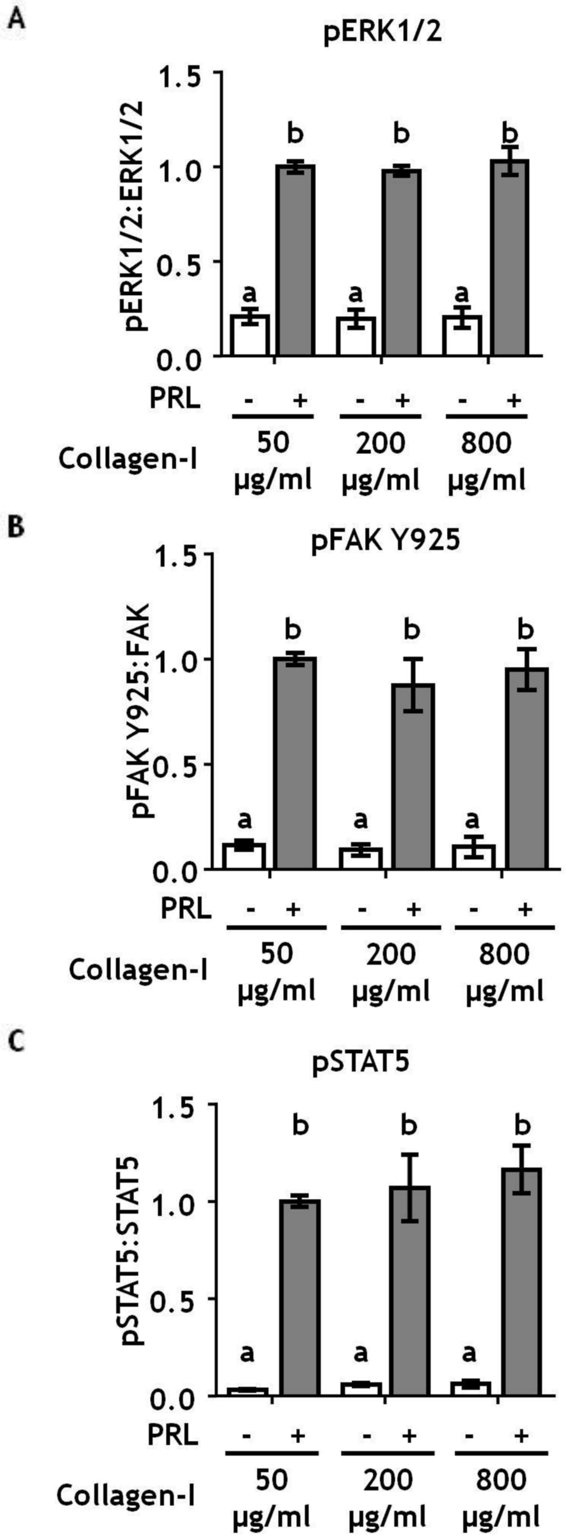

Supplementary Figure S3: Compliant stiffness rescues collagen-I ligand density PRL-induced signals to pSTAT5. A-C. T47D were cells plated on 12 kPa polyacrylamide gels coated with either 50, 200, or 800 µg/ml collagen-I, serum starved for 24h, then treated ± PRL (4 nM) for 15 min. Cell lysates were immunoblotted with the indicated antibodies. *Top panels:* Representative immunoblots. *Bottom panels:* Quantification of immunoblots by densitometry. Means ± S.E.M. n = 3. Different letters represent significant differences between treatments, $p<0.05$.
